# Preoperative Metabolic Predictors of Granulation Subtypes in Somatotroph Tumors: A Multicenter Retrospective Cohort Study

**DOI:** 10.1002/cns.70774

**Published:** 2026-02-03

**Authors:** Le Chen, Jiaming Wang, Ailiang Zeng, Farhana Akter, Shanshan Wang, Shitong Liu, Weiyu Hu, Shun Yao, Konstantinos Margetis, Zongming Wang, Haipeng Liu, Xin Wang

**Affiliations:** ^1^ Department of Neurosurgery The First Affiliated Hospital of Guangdong Pharmaceutical University Guangzhou Guangdong China; ^2^ School of Basic Medical Sciences, Guangdong Pharmaceutical University Guangzhou Guangdong China; ^3^ Center for Pituitary Tumor Surgery, Department of Neurosurgery The First Affiliated Hospital, Sun Yat‐Sen University Guangzhou Guangdong China; ^4^ Department of Neurosurgery Icahn School of Medicine at Mount Sinai New York New York USA; ^5^ Faculty of Arts and Sciences, Harvard University Cambridge Massachusetts USA; ^6^ Department of Neurosurgery Pituitary Tumor Center, The Sixth Affiliated Hospital, Sun Yat‐Sen University Guangzhou Guangdong China; ^7^ National Medical Research Association Leicester UK; ^8^ Centre for Intelligent Healthcare, Coventry University Coventry UK; ^9^ Cardiovascular Analytics Group Hong Kong SAR China; ^10^ Universidad Santa Paula Curridabat San José Costa Rica

**Keywords:** granulation subtypes, machine learning, somatotroph tumors, triglyceride, uric acid

## Abstract

**Purpose:**

Differentiating between sparsely granulated and densely granulated somatotroph tumors (SGSTs and DGSTs) currently relies on postoperative immunohistochemistry. This study aimed to evaluate whether triglyceride (TG), uric acid (UA), and their composite TG–UA index [ln(TG × 1000/UA)] could serve as preoperative indicators for distinguishing granulation subtypes of somatotroph tumors.

**Methods:**

In this multicenter retrospective cohort study, 230 patients with somatotroph tumors were analyzed. Logistic regression and generalized additive models assessed associations and potential nonlinear associations between metabolic indicators and granulation subtypes. Predictive performance was compared between models using UA and TG separately and those using the TG–UA index.

**Results:**

SGSTs were associated with significantly higher TG, growth hormone, insulin‐like growth factor 1, and TG–UA index values. The TG–UA index remained an independent predictor of the SGST subtype (OR = 1.514, *p* = 0.014). Predictive performance was similar between models (*p* = 0.108).

**Conclusion:**

The TG–UA index is a promising noninvasive biomarker for identifying the SGST subtype in somatotroph tumors. Although limited by its retrospective design and lack of long‐term data, this study provides a foundation for future prospective validation.

## Graphical abstract

Somatotroph tumors, a rare subtype of pituitary adenomas, can be histologically classified into sparsely granulated (SGSTs) and densely granulated somatotroph tumors (DGSTs) based on cytokeratin staining. SGSTs are generally more invasive and less responsive to medical therapy, making their early identification clinically important. This study identified an independent association between the TG–UA index and granulation subtypes and, for the first time, proposed the TG–UA index [ln((TG × 1000)/UA)] as a novel, noninvasive, and cost‐effective biomarker for the preoperative differentiation of SGSTs from DGSTs.

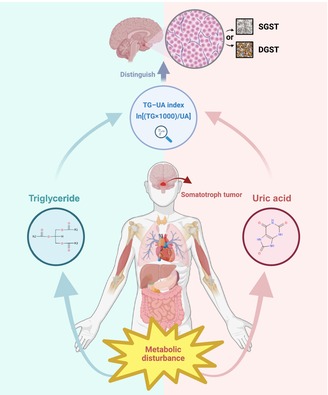

## 1 Introduction

Pituitary neuroendocrine tumors (PitNETs) are common tumors in the sellar region and are typically classified as functioning and nonfunctioning tumors based on hormone secretion [1]. Somatotroph tumors, a subtype of functioning PitNETs, are the leading cause of acromegaly in adults and gigantism in children [1, 2]. Histologically, somatotroph tumors are further classified into sparsely granulated and densely granulated somatotroph tumors (SGSTs and DGSTs), based on cytokeratin staining patterns. SGSTs are generally more invasive and less responsive to medical treatment [3, 4]. However, classification still depends on postoperative immunohistochemistry, emphasizing the need for reliable, noninvasive preoperative biomarkers.

Metabolic dysregulation is a well‐known feature of somatotroph tumors, primarily due to chronic activation of the growth hormone (GH)/insulin‐like growth factor 1 (IGF‐1) axis [5, 6]. GH excess promotes insulin resistance and altered energy substrate metabolism, resulting in elevated serum levels of triglyceride (TG) and uric acid (UA) [7, 8]. Notably, UA concentrations correlate with IGF‐1 levels and decrease after surgical remission, supporting its potential as a surrogate marker of disease activity [9]. While TG and UA reflect different aspects of metabolic dysfunction, their combined predictive utility for subtyping somatotroph tumors remains unexplored. In other endocrine and cardiometabolic conditions, composite metabolic indices, such as the triglyceride–glucose index and the atherogenic index of plasma, are used to assess integrated metabolic risk through logarithmic transformation of ratio‐based markers [10, 11, 12]. However, no studies have specifically assessed whether a composite index integrating TG and UA can differentiate somatotroph tumor granulation subtypes preoperatively. This represents a significant gap in the current literature.

In this study, we evaluated TG and UA as individual markers and proposed their composite form—the triglyceride–uric acid (TG–UA) index, calculated as ln[(TG × 1000)/UA]—as a potential noninvasive biomarker to distinguish SGSTs from DGSTs. We hypothesized that this composite index could reflect granulation subtype–specific metabolic phenotypes and provide clinical utility for individualized preoperative stratification and treatment planning.

## 2 Methods

### 2.1 Study Design and Data Source

This multicenter, retrospective cohort study was conducted at three hospitals affiliated with Sun Yat‐sen University: the First Affiliated Hospital (Center A), the Sun Yat‐sen University Cancer Center (Center B), and the Huangpu Branch of The First Affiliated Hospital (Center C). From November 2019 to June 2024, 281 consecutive patients who underwent transsphenoidal surgery for somatotroph tumors were screened.

Patients were included if they met the following criteria: (i) histopathological confirmation of a somatotroph tumor; (ii) nadir GH level > 1 μg/L during a 75‐g oral glucose tolerance test; (iii) elevated IGF‐1 levels; and (iv) no use of medications known or suspected to affect lipid or UA metabolism at the time of admission. Exclusion criteria were: (i) missing data on tumor granulation subtype; (ii) absence of preoperative magnetic resonance imaging; and (iii) > 30% missing data in variables.

**FIGURE 1:**
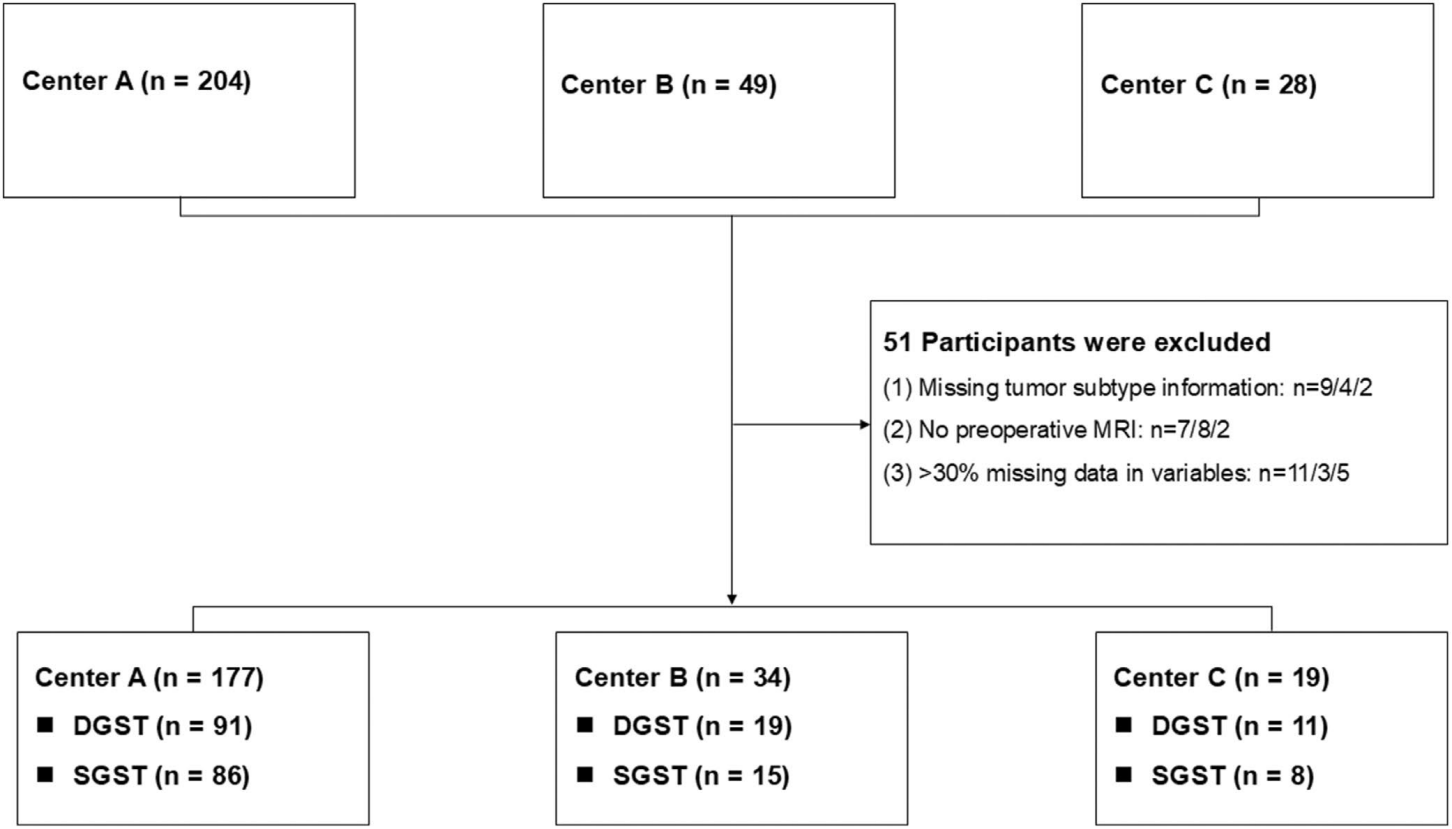
Flowchart of participant selection. MRI, magnetic resonance imaging.

After applying the eligibility criteria, anonymizing patient records, and performing standardized preprocessing, 230 patients were included in the final analysis (Figure 1). The primary outcome was the granulation subtype of somatotroph tumors, classified as SGST (coded as 1) or DGST (coded as 0). Key predictors included serum TG, UA, and the composite TG–UA index.

### 2.2 Calculation of Triglyceride–Uric Acid Index

The TG–UA index was calculated as the natural logarithm of the ratio between TG (mmol/L) and UA (μmol/L), with TG multiplied by 1000 to harmonize units:

$$\mathrm{TG}-\mathrm{UA}\;\text{index}=\ln\left(\frac{\mathrm{TG}\times 1000}{\mathrm{UA}}\right)$$

This ratio‐based structure integrates information from two metabolic markers into a single composite index, allowing for a more comprehensive assessment of metabolic status. Similar ratio‐based indices, such as the uric acid‐to‐high‐density lipoprotein cholesterol ratio and other comparable composite markers, have been widely used in metabolic research [13, 14, 15]. A logarithmic transformation was applied for two main purposes: (i) to stabilize variance by reducing the influence of extreme values [16, 17], and (ii) to convert proportional changes in the TG‐to‐UA ratio into approximately additive, linear changes in the index, thereby improving statistical robustness and interpretability [18, 19] (Figure S1; Table S1). This transformation is consistent with established methods for constructing composite metabolic indices, such as the triglyceride–glucose index and the atherogenic index of plasma [10, 11, 12].

### 2.3 Pathological Assessment

Granulation subtypes of somatotroph tumors were identified by immunohistochemical (IHC) staining using the anti‐cytokeratin antibody CAM5.2. Tumor specimens were fixed in 4% neutral‐buffered formalin for 24 h, embedded in paraffin, and sectioned at a thickness of 6 μm. Initial hematoxylin and eosin staining was performed to confirm the diagnosis of a pituitary tumor. Subsequent IHC staining was conducted using antibodies against CAM5.2, somatostatin receptor 2, Ki‐67, pituitary hormones (GH, prolactin, follicle‐stimulating hormone), and transcription factors (PIT‐1, SF‐1, and T‐PIT).

All slides were independently evaluated under light microscopy by two experienced pathologists blinded to the clinical data. Any discrepancies in classification were resolved through consensus. CAM5.2 staining patterns were categorized based on the criteria proposed by Obari et al. [20], as follows: (i) perinuclear (ringlike) pattern: no staining, < 70%, or ≥ 70% positive cells; (ii) dot‐like pattern (fibrous bodies): no staining, 1%–8%, 9%–69%, or ≥ 70% positive cells; (iii) transitional pattern: no staining, < 70%, or ≥ 70% positive cells. Tumors exhibiting a perinuclear pattern in ≥ 70% of cells or a dot‐like pattern in ≤ 8% were classified as DGSTs, whereas those with a dot‐like pattern in ≥ 70% were defined as SGSTs.

### 2.4 Covariates

Covariates were selected based on prior studies and their established clinical relevance to the classification of somatotroph tumor subtypes [9, 21, 22, 23, 24, 25, 26, 27]. The following variables were included: (i) categorical variables: sex, hypertension, diabetes, Knosp grade, and center; (ii) continuous variables: age, body mass index (BMI), duration of disease, TG, UA, total cholesterol (TC), low‐density lipoprotein cholesterol (LDL‐C), GH, IGF‐1, tumor volume, and maximum tumor diameters (transverse, anteroposterior, and superoinferior) (Materials S1; Table S2).

### 2.5 Missing Data Processing

**FIGURE 2:**
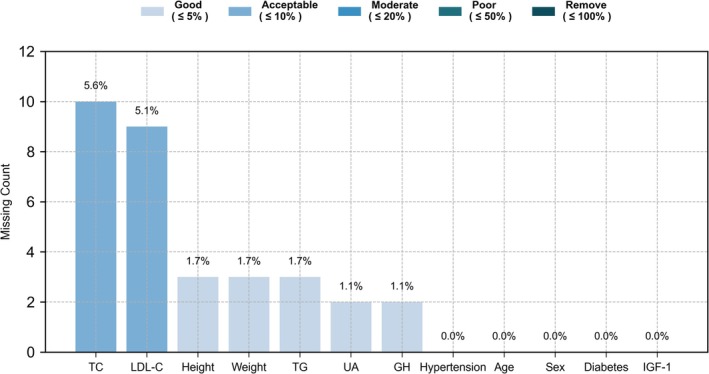
Visualization of missing data patterns for selected variables in Center A. Bars represent the number of missing observations for each variable, and the values atop the bars indicate the proportion of missing data. GH, growth hormone; IGF‐1, insulin‐like growth factor 1; LDL‐C, low‐density lipoprotein cholesterol; TC, total cholesterol; TG, triglyceride; UA, uric acid.

Given the observed missingness patterns (Figure 2), complete‐case analysis would have reduced statistical power and introduced potential selection bias. Therefore, missing data were imputed using multiple imputation by chained equations to preserve data integrity [28] (Materials S2).

### 2.6 Statistical Analyses

All statistical analyses were conducted using Python (version 3.9.18) and R (version 4.3.1). All statistical tests were two‐sided, and *p* < 0.05 was considered statistically significant.

Baseline characteristics between DGSTs and SGSTs were compared using appropriate statistical tests based on variable type. Normality of continuous variables was assessed using the Shapiro–Wilk test, and homogeneity of variance was assessed using Levene's test. Variables with normal distributions and equal variances were compared using Student's t‐test; those with unequal variances were compared using Welch's *t*‐test; and non‐normally distributed variables were compared using the Mann–Whitney *U* test. Categorical variables were compared using the chi‐square test or Fisher's exact test, as appropriate.

To investigate the associations of TG, UA, and the TG–UA index with tumor granulation subtype, univariate and multivariate logistic regression models were constructed using three adjustment strategies: (i) Model I: unadjusted; (ii) Model II: adjusted for age, sex, and center; and (iii) Model III: further adjusted for BMI, hypertension, diabetes, TC, LDL‐C, GH, IGF‐1, Knosp grade, and other potential confounders. Multicollinearity was assessed using Pearson correlation coefficients and the variance inflation factor (VIF). Highly collinear variables were addressed using principal component analysis (PCA) [29, 30] (Materials S3).

Generalized additive models (GAMs) with a logistic link were applied to examine potential nonlinear associations between continuous predictors and the log‐odds of granulation subtype. GAMs allowed flexible modeling of nonlinear associations between predictors and outcomes via smoothing splines.

Subgroup analyses were conducted by stratifying the data by sex, median age, hypertension, and diabetes. Logistic regression models in each subgroup were adjusted for key covariates, excluding the stratifying variable. Interaction effects were examined in the overall cohort using likelihood ratio tests.

### 2.7 Evaluation of the TG–UA Index as a Substitute for Individual UA and TG Predictors

To evaluate whether the TG–UA index could serve as a substitute for the joint effects of TG and UA, we compared two modeling strategies: one that included TG and UA as separate covariates (TG+UA model), and another that replaced them with their composite index (TG–UA model), keeping all other predictors constant.

Multiple machine learning algorithms were assessed using cross‐validation on the training cohort (Center A). The best‐performing model, identified by the area under the receiver operating characteristic curve (AUC) from cross‐validation on the training cohort, was retrained on the entire training cohort. External validation was conducted on the external validation cohort (Centers B and C), with predictor variables standardized using parameters estimated from the training cohort. Model performance was compared using DeLong's test to assess differences in AUC (Materials S4).

## 3 Results

### 3.1 Participant Characteristics

A total of 177 patients with somatotroph tumors were enrolled from Center A (91 DGSTs and 86 SGSTs). After combining data from Centers A–C, the final study cohort consisted of 230 patients (121 DGSTs and 109 SGSTs) (Table 1; Table S3). For example, sex, age, BMI, duration of disease, hypertension, and diabetes were comparable between the DGST and SGST groups. Metabolically, patients with SGSTs showed higher TG levels and TG–UA index values, accompanied by a trend toward lower UA levels. Endocrine evaluation revealed significantly higher GH and IGF‐1 levels in the SGST group. Radiologically, the SGST group demonstrated larger maximum tumor diameters (transverse, anteroposterior, and superoinferior) and tumor volume, whereas the Knosp grade distribution was similar between the two groups.

**TABLE 1:** Comparison of baseline characteristics between DGST and SGST patients in Center A and the combined multicenter cohort (Centers A–C).

Variables	A		*p*	A–C		*p*
	DGST (*n* = 91)	SGST (*n* = 86)		DGST (*n* = 121)	SGST (*n* = 109)	
Demographic characteristics						
Sex						
Male	48 (52.7%)	39 (45.3%)	0.404	64 (52.9%)	46 (42.2%)	0.137
Age	42.0 (33.0, 52.0)	37.0 (31.0, 49.0)	0.129	42.0 (33.0, 52.0)	38.0 (31.0, 49.0)	0.156
BMI	25.2 (23.2, 26.8)	25.1 (23.3, 26.3)	0.861	25.2 (23.1, 27.0)	24.9 (23.2, 26.2)	0.520
Metabolic and hormonal features						
UA	358.5 ± 94.4	327.7 ± 90.4	0.028	353.6 ± 96.2	329.8 ± 89.7	0.055
TG	1.3 (0.8, 1.7)	1.6 (1.2, 2.3)	< 0.001	1.3 (0.9, 1.9)	1.5 (1.1, 2.3)	0.003
LDL‐C	2.8 (2.2, 3.2)	2.9 (2.5, 3.4)	0.046	2.8 (2.3, 3.2)	2.8 (2.5, 3.3)	0.223
TG–UA index	1.2 (0.9, 1.7)	1.7 (1.2, 2.0)	< 0.001	1.3 (0.9, 1.7)	1.6 (1.2, 2.0)	< 0.001
GH	8.8 (2.4, 19.6)	15.0 (4.3, 27.3)	0.037	9.0 (2.7, 21.1)	15.2 (4.4, 26.1)	0.037
IGF‐1	470.0 (341.5, 540.5)	502.5 (417.8, 615.0)	0.012	479.0 (362.0, 556.0)	504.0 (437.0, 609.0)	0.017
Tumor characteristics						
Maximum transverse diameter	15.0 (11.0, 21.0)	19.6 (14.4, 27.0)	0.003	16.0 (11.0, 21.0)	20.0 (14.7, 27.0)	< 0.001
Maximum anteroposterior diameter	14.0 (10.0, 18.0)	17.0 (13.0, 20.0)	0.023	14.0 (11.0, 18.0)	16.0 (13.0, 20.0)	0.009
Maximum superoinferior diameter	14.0 (9.0, 20.0)	17.5 (10.9, 30.8)	0.026	15.0 (10.0, 21.5)	18.0 (12.8, 31.0)	0.006
Tumor volume	1.7 (0.4, 4.4)	2.9 (1.0, 7.8)	0.008	1.7 (0.4, 4.5)	2.9 (1.3, 7.9)	0.002

Abbreviations: A, Center A; A–C, Centers A–C; BMI, body mass index; GH, growth hormone; IGF‐1, insulin‐like growth factor 1; LDL‐C, low‐density lipoprotein cholesterol; TG, triglyceride; UA, uric acid.

### 3.2 Regression Analysis

**FIGURE 3:**
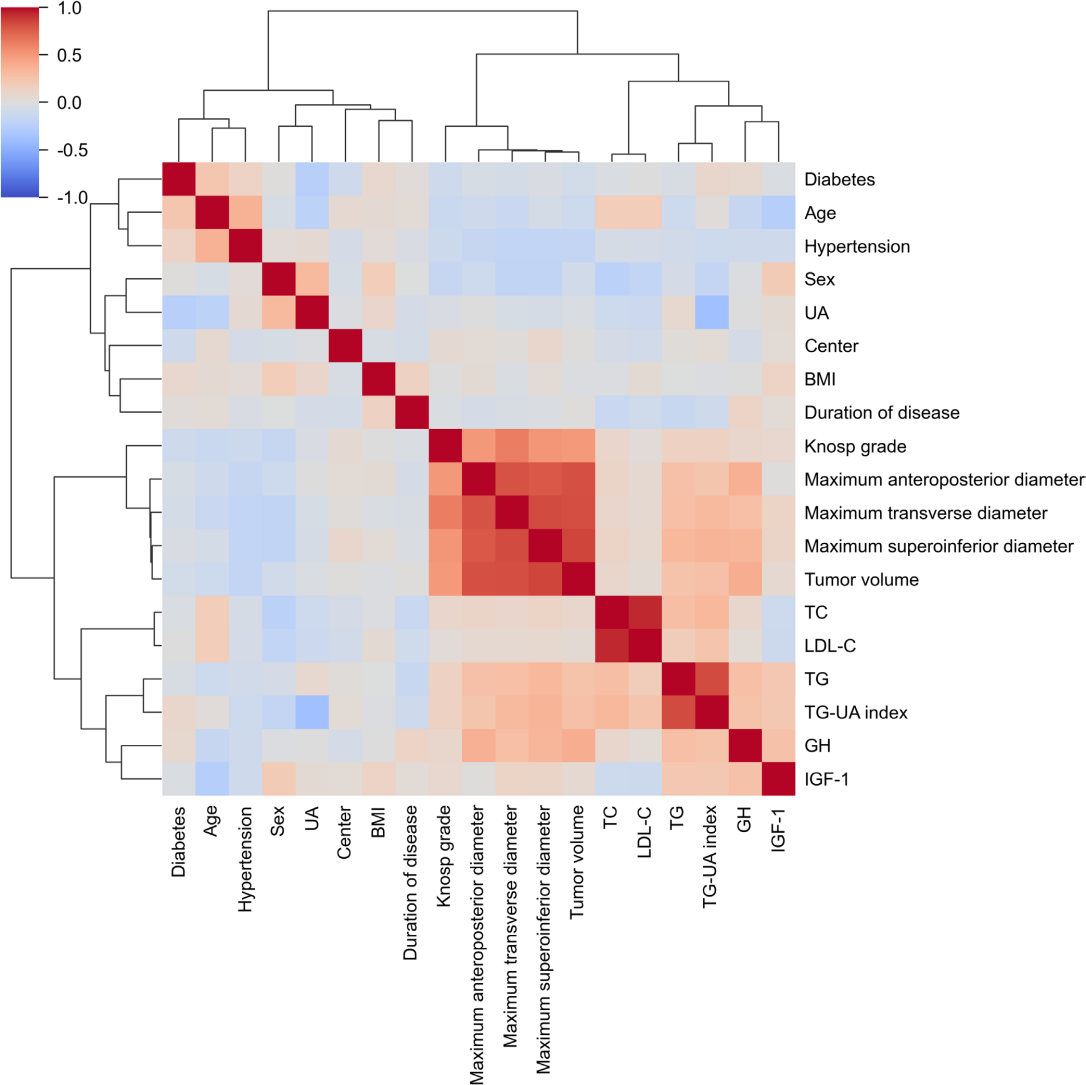
Pearson correlation heatmap of study variables. GH, growth hormone; IGF‐1, insulin‐like growth factor 1; LDL‐C, low‐density lipoprotein cholesterol; TC, total cholesterol; TG, triglyceride; UA, uric acid.

Pairwise Pearson correlation coefficients (Figure 3) and VIF analyses revealed substantial multicollinearity between TC and LDL‐C, as well as among tumor volume and the maximum tumor diameters. To address collinearity, PCA was applied separately to each group of highly correlated variables (Table S4).

**FIGURE 4:**
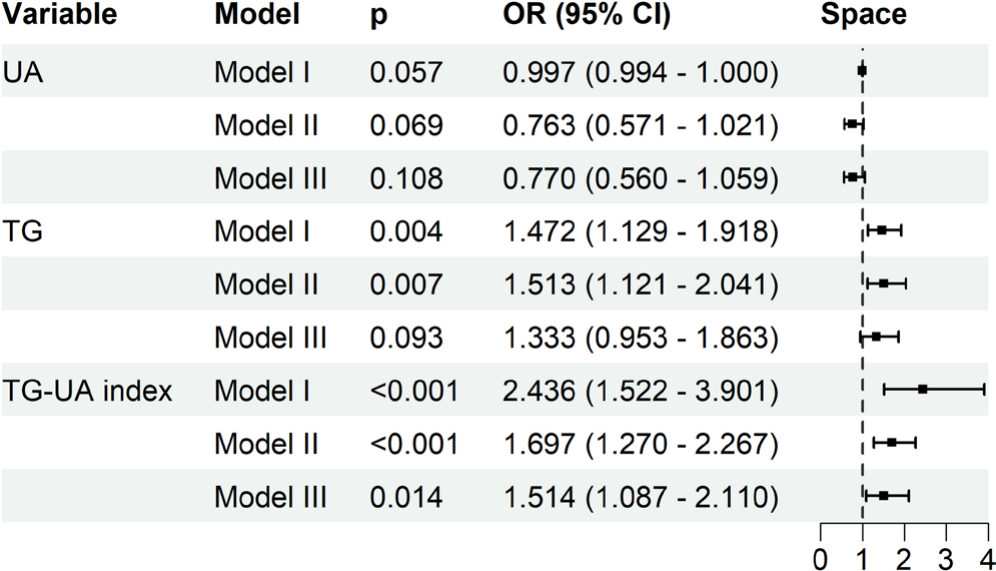
Forest plot of associations of TG, UA, and the TG–UA index with the sparsely granulated subtype across different regression models. 95% CI, 95% confidence interval; OR, odds ratio; TG, triglyceride; UA, uric acid.

The results of the logistic regression analyses are summarized (Figure 4). In the fully adjusted model (Model III), the TG–UA index remained significantly and independently associated with the SGST subtype (OR = 1.514; 95% CI: 1.087–2.110; *p* = 0.014). In contrast, TG exhibited a positive association in unadjusted models but lost statistical significance after covariate adjustment. UA alone showed no significant association with the SGST subtype in any model.

### 3.3 Nonlinear Association Analysis

GAMs identified significant nonlinear associations of TG (*p* = 0.013) and the TG–UA index (*p* = 0.021), whereas UA showed no significant nonlinear association (*p* = 0.525).

### 3.4 Subgroup Analysis

The associations of TG, UA, and the TG–UA index with granulation subtypes were consistent across predefined subgroups (Table S5). Although certain within‐group associations reached statistical significance, the tests for interaction were not significant (all *p* > 0.05), indicating no evidence of effect modification. The direction and magnitude of these associations remained consistent across sex, median age, and metabolic comorbidity status. These findings further support the robustness of TG and the TG–UA index as reliable metabolic markers for differentiating granulation subtypes of somatotroph tumors.

### 3.5 Utility of the TG–UA Composite Variable

**FIGURE 5:**
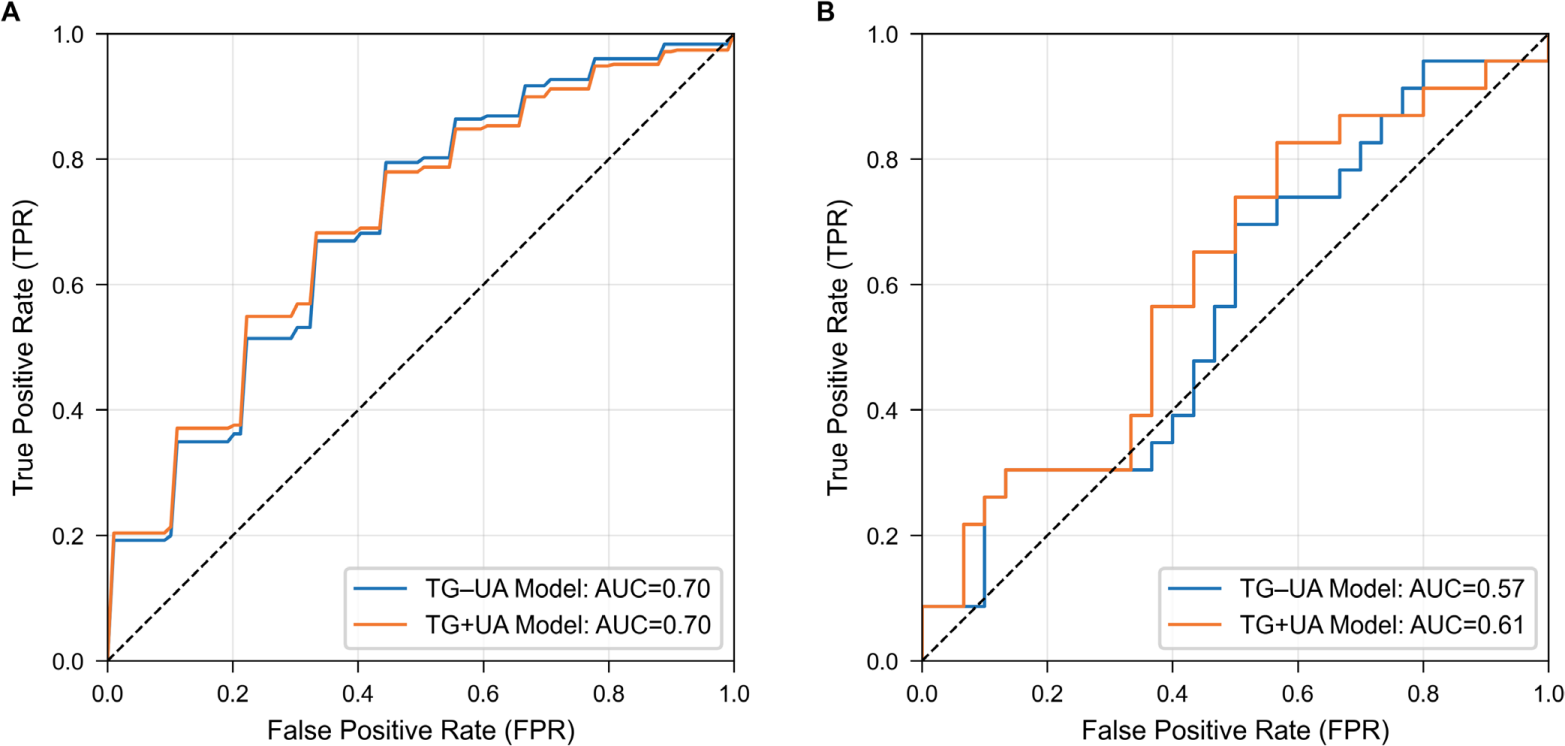
ROC curves comparing TG + UA and TG–UA Index models for somatotroph tumors subtyping in the training cohort from Center A (A) and external validation cohort from Centers B and C (B).

Feature selection using both the Boruta algorithm and the least absolute shrinkage and selection operator (LASSO) consistently identified TG, UA, the TG–UA index, IGF‐1, and maximum transverse diameter as key predictors. Two predictive models were developed: (i) TG+UA model, which included TG, UA, IGF‐1, and maximum transverse diameter using logistic regression; (ii) TG–UA index model, which replaced TG and UA with the TG–UA index while retaining IGF‐1 and maximum transverse diameter, developed using linear discriminant analysis. In the training cohort, both models demonstrated similar discriminative performance, with closely matched AUCs, accuracy, sensitivity, and specificity (Table 2). In the external validation cohort, both models maintained comparable performance (Table 2); DeLong's test revealed no significant difference in AUCs (*p* = 0.108). The receiver operating characteristic (ROC) curves of the TG + UA and TG–UA models were broadly similar in both the training and external validation cohorts (Figure 5).

**TABLE 2:** Comparison of predictive performance between TG+UA and TG–UA models for GHPA subtyping in the training and external validation cohorts.

Cohort	Model	AUC	Accuracy	Specificity	Sensitivity
Training cohort	TG–UA Model	0.70 (0.67, 0.72)	0.65 (0.63, 0.67)	0.68 (0.65, 0.71)	0.62 (0.59, 0.65)
	TG+UA Model	0.70 (0.67, 0.72)	0.65 (0.63, 0.67)	0.68 (0.66, 0.71)	0.61 (0.58, 0.65)
External validation cohort	TG–UA Model	0.57	0.55	0.50	0.61
	TG+UA Model	0.61	0.57	0.50	0.65

Abbreviations: AUC, area under the receiver operating characteristic curve; External validation cohort, Centers B and C; Training cohort, Center A.

## 4 Discussion

In this multicenter retrospective study, we systematically investigated the associations of TG, UA, and their composite marker—the TG–UA index—with somatotroph tumor granulation subtypes. Patients with SGSTs had higher TG, IGF‐1, and GH levels, larger maximum tumor diameters and tumor volume, and elevated TG–UA index values compared to those with DGSTs. Multivariate logistic regression analyses confirmed that the TG–UA index was an independent predictor of SGST subtype, consistent across all adjustment models. GAMs further demonstrated significant nonlinear associations of TG and the TG–UA index, while UA showed no evidence of a nonlinear association. Subgroup analyses stratified by sex, age, hypertension, and diabetes revealed consistent patterns, indicating that the observed associations were not solely attributable to specific confounders.

Notably, feature selection using both the Boruta algorithm and LASSO independently identified the same subset of predictors: TG, UA, the TG–UA index, IGF‐1, and maximum transverse diameter. This convergence underscores their potential importance in differentiating granulation subtypes. Furthermore, predictive models incorporating either the TG–UA index or the individual TG and UA predictors showed similar classification performance, supporting the TG–UA index as a simplified yet clinically meaningful surrogate.

These findings are consistent with prior evidence linking the GH/IGF‐1 axis to disturbances in lipid and purine metabolism. Previous studies have shown that UA levels correlate with IGF‐1 during active disease and decrease following surgical remission [9]. Additionally, GH abnormalities have been linked to hyperuricemia and lipid disturbances in diverse populations [8]. GH excess promotes hepatic TG synthesis and lipolysis, contributing to the elevated TG levels observed in somatotroph tumors [31]. Thus, the TG–UA index may reflect integrated metabolic dysregulation characteristic of SGSTs, providing biological plausibility for its predictive value.

Despite its strengths, this study has several limitations. First, the retrospective design and moderate sample size (*n* = 230) limit causal inference and the generalizability of the findings. Second, all patients were recruited from tertiary centers in southern China, which may introduce geographic and ethnic bias. Third, the absence of postoperative or longitudinal follow‐up prevents evaluation of the TG–UA index in monitoring treatment outcomes. Finally, although this study focused on the TG–UA index, it did not directly compare its predictive performance with that of other established composite metabolic markers, such as the triglyceride–glucose index or the atherogenic index of plasma [32, 33].

Future studies should aim to validate these findings in larger and more ethnically diverse populations, and to evaluate the comparative utility of the TG–UA index alongside other metabolic indices. Integrating this marker into multimodal predictive frameworks that incorporate imaging, clinical, and molecular data may enhance preoperative stratification and support personalized management of somatotroph tumors.

## 5 Conclusion

This study identified independent associations of TG and the TG–UA index with somatotroph tumor granulation subtypes and, for the first time, proposed the TG–UA index as a novel, noninvasive, and cost‐effective preoperative biomarker for predicting the SGST subtype. The TG–UA index offers both simplicity and clinical utility, with predictive performance comparable to models that incorporate TG and UA as separate predictors. Integrating the TG–UA index with radiological and molecular data may facilitate the development of more accurate predictive models, ultimately enhancing surgical decision‐making, individualized treatment planning, and long‐term management in patients with somatotroph tumors.

## Author Contributions

L.C., S.Y., and H.L.: conceptualization and design. L.C., J.W., and A.Z.: writing – original draft preparation; H.L., A.Z., S.Y., K.M., Z.W., and X.W.: writing – reviewing and editing. L.C., F.A., S.W., and S.L.: formal analysis. L.C., J.W., A.Z., F.A., W.H., K.M., H.L., and X.W.: results interpretation. L.C. and S.L.: visualization. K.M., Z.W., and H.L.: supervision. S.Y., L.C., and J.W.: acquisition of data. All authors read and approved the final manuscript.

## Funding

This work was supported by the Guangdong Province Key Technologies R&D Program for “Brain Science and Brain‐like Intelligence Research” (2023B0303020002), Guangdong Basic and Applied Basic Research Foundation (2024A1515011697), Guangdong Province Administration of Traditional Chinese Medicine Project (20231210), Key Clinical Technique of Guangzhou (2023P‐ZD18), and Guangdong Medical Association Clinical Research Special Fund (No. 2024HY‐A6003).

## Ethics Statement

This study was approved by the Institutional Review Board and Ethics Committee of The First Affiliated Hospital, Sun Yat‐sen University. Given its retrospective nature, the requirement for informed consent was waived by the ethics committee, and all patient data were anonymized prior to analysis to protect patient privacy.

## Conflicts of Interest

The authors declare no conflicts of interest.

## Supporting information


**Materials S1.** Detailed description of variables, coding schemes, and measurement protocols.
**Materials S2**. Details of missing data imputation.
**Materials S3**. Multicollinearity diagnostics and dimensionality reduction.
**Materials S4**. Predictive modeling detail.

**Table S1:** Summary statistics of the raw TG/UA ratio and its log‐transformed TG–UA index.
**Table S2:** Detailed description of variables and coding schemes.
**Table S3:** Comparison of DGST and SGST patients in Center A and the combined cohort (Centers A–C).
**Table S4:** Principal component loadings and explained variance for lipid and tumor morphology variables.
**Table S5:** Subgroup analysis of UA, TG, and the TG–UA Index.

## Data Availability

The datasets generated during and/or analyzed during the current study are available from the corresponding author on reasonable request.
