# Supplementary material for: Preoperative Metabolic Predictors of Granulation Subtypes in Somatotroph Tumors: A Multicenter Retrospective Cohort Study
Source: CNS Neurosci Ther. 2026 Feb 3;32(2):e70774. doi: 10.1002/cns.70774 (PMC12865497; doi:10.1002/cns.70774)
Supplement: Supplementary file 1 — Materials S1. Detailed description of variables, coding schemes, and measurement protocols. Materials S2. Details of missing data imputation. Materials S3. Multicollinearity diagnostics and dimensionality reduction. Materials S4. Predictive modeling detail. [file CNS-32-e70774-s001.docx]

**Pre-operative Metabolic Predictors of Granulation Subtypes in Somatotroph Tumors: A Multicenter Retrospective Cohort Study**

***Supplementary Materials***

**Supplementary Material S1. Detailed Description of Variables, Coding Schemes, and Measurement Protocols**

1. Variable Definitions and Coding Schemes

All variables used in the analyses are summarized in Supplementary Table S2, together with their variable types (categorical vs continuous), coding schemes, and measurement units.

1. Imaging Assessment

Imaging parameters—including tumor volume, maximum tumor diameters (transverse, anteroposterior, and superoinferior), and Knosp grade—were evaluated independently by two experienced neurosurgeons. Any discrepancies were resolved through consensus to ensure data accuracy and reproducibility.

1. Metabolic Marker Measurement and Patient Preparation

All metabolic markers were quantified using fasting venous blood samples collected at 8:00 AM on the morning of the second day of hospitalization. To ensure measurement accuracy, patients were strictly instructed to fast from 10:00 PM the preceding night to minimize the influence of dietary intake on metabolic parameters. Furthermore, patients receiving long-term medications for hyperlipidemia, hypercholesterolemia, or hyperuricemia at the time of admission were excluded to avoid potential confounding effects.

**Supplementary Material S2. Details of Missing Data Imputation**

Missing data were assumed to be missing at random and were handled using the multiple imputation by chained equations approach, implemented via the IterativeImputer class in scikit-learn (version 1.4.2) [1]. The algorithm modeled each variable with missing values as a function of other variables in a round-robin fashion, with a maximum of 10 iterations set per imputation cycle to ensure convergence.

To assess the validity of imputation, we compared the distributions of covariates before and after imputation using the Kolmogorov–Smirnov test [2]. No statistically significant differences were detected (all p > 0.05), indicating that the imputation process preserved the original distributional characteristics without introducing systematic bias.

**Supplementary Material S3****. Multicollinearity Diagnostics and Dimensionality Reduction**

In Model III, a systematic approach was employed to assess and address multicollinearity. Pearson correlation coefficients were initially computed to identify highly correlated variable pairs [3], which were further assessed using the variance inflation factor (VIF). For variables with VIF > 10 [4], principal component analysis was applied to reduce dimensionality while retaining most of the original variance [5]. Principal components were retained until the cumulative explained variance exceeded 85%. These orthogonal components were subsequently incorporated into the final multivariate logistic regression model, substituting the original collinear variables [6].

**Supplementary Material S4. Predictive Modeling Details**

To evaluate whether the TG–UA index could serve as a substitute for the joint effects of TG and UA, we designed and compared two predictive modeling strategies. One model included TG and UA as separate covariates (TG+UA model), while the other replaced them with the TG–UA index (TG–UA model), with an identical set of other covariates maintained.

1. Model Candidates and Implementation

Multiple machine learning algorithms were implemented and compared to develop predictive models for differentiating SGSTs from DGSTs [7].

The candidate algorithms included the following:

1. Logistic Regression
2. Linear Discriminant Analysis
3. Multilayer Perceptron
4. K-Nearest Neighbors
5. Support Vector Machine
6. Naive Bayes
7. Extreme Gradient Boosting
8. Random Forest
9. Feature Selection

A robust two-step feature selection strategy was adopted to identify the most informative predictors:

1. Boruta Algorithm: The Boruta algorithm was first applied to screen all potentially relevant predictors [8]. This wrapper method iteratively compares the importance of real features with that of randomized shadow features using a random forest classifier. In this study, the algorithm was run for 100 iterations, and features confirmed as “important” were retained.
2. LASSO Regression: Least Absolute Shrinkage and Selection Operator (LASSO) regression was then used to further refine the variable set [9]. LASSO performs variable selection by imposing an L1 penalty on regression coefficients to induce sparsity. The optimal penalty parameter (λ) was determined via 5-fold cross-validation (CV = 5) to prevent overfitting and enhance generalizability.

The final predictor set was obtained by taking the intersection of features selected by both Boruta and LASSO.

1. Hyperparameter Tuning

To optimize model performance, hyperparameters were tuned using the Optuna framework [10]. The optimal configuration for each model was identified based on the mean area under the receiver operating characteristic curve (AUC) across cross-validation folds, and the model with the highest mean AUC was selected for external validation.

1. Evaluation Metrics

The performance of all models was evaluated using both discrimination and classification metrics:

1. Primary metric:
   1. AUC: Reflects the model’s global ability to discriminate between SGST and DGST subtypes.
2. Additional metrics:
   1. Accuracy: The proportion of correctly classified samples.
   2. Sensitivity: The true positive rate — the proportion of SGSTs correctly identified as SGST subtype.
   3. Specificity: The true negative rate — the proportion of DGSTs correctly identified as DGST subtype.

To ensure statistical robustness, 95% confidence intervals for all evaluation metrics (AUC, accuracy, sensitivity, and specificity) were calculated using bootstrap resampling (1,000 resamples).

***Supplementary Figures***


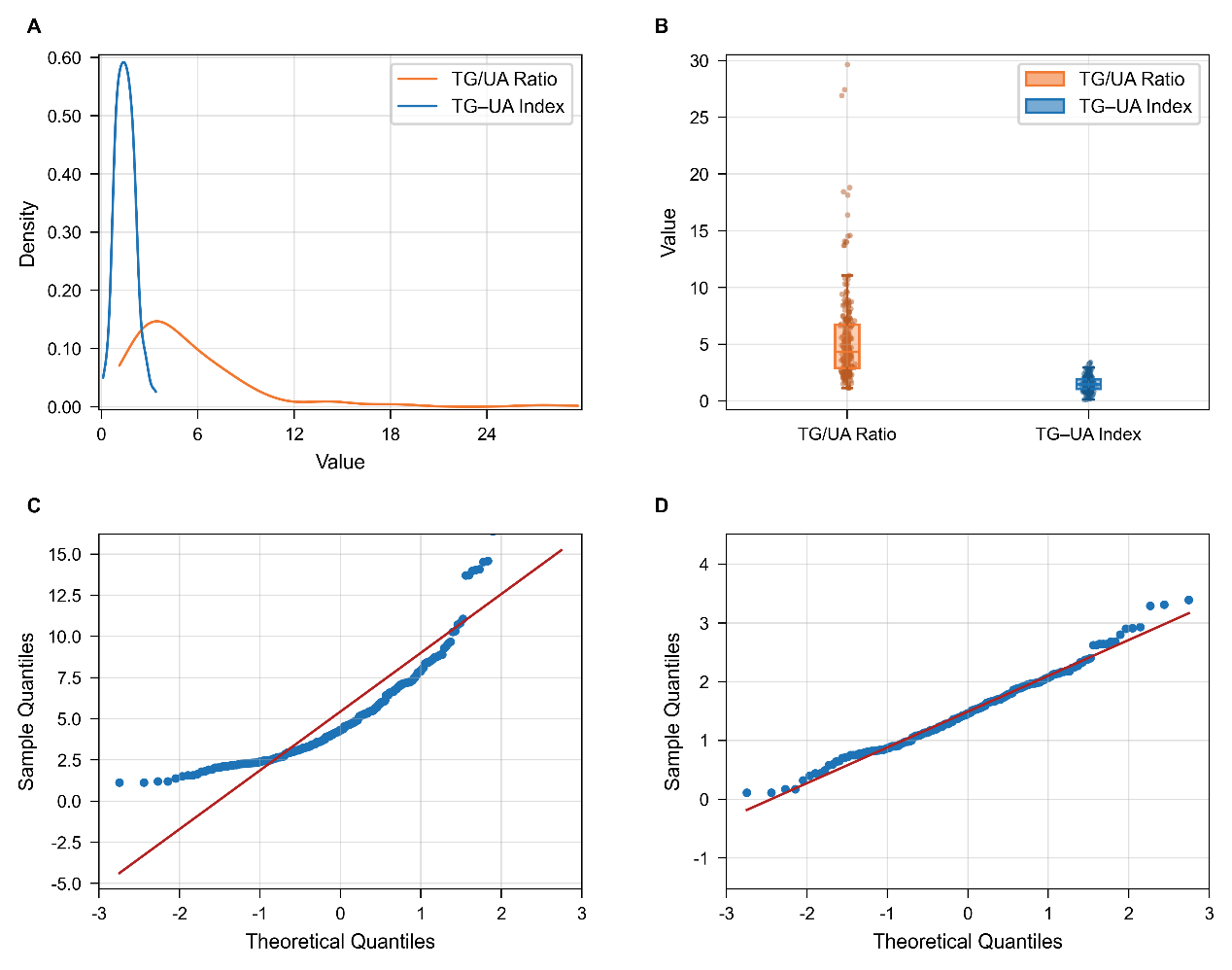


Abbreviations: TG, triglyceride; UA, uric acid.

**Supplementary Figure S1. Distribution of the raw TG/UA ratio and its log-transformed TG–UA index**

(A) Overlaid kernel density estimates for the TG/UA ratio and its log-transformed TG–UA index. The curves represent two metrics (raw ratio vs log-transformed index) and are overlaid to compare distributional shape rather than absolute values. The TG–UA index exhibits a more concentrated distribution with a markedly reduced right tail, consistent with variance stabilization and reduced influence of extreme values.
(B) Side-by-side boxplots with overlaid jittered points comparing the TG/UA ratio and its log-transformed TG–UA index. The raw ratio shows a broader interquartile range and more high-end outliers, whereas the log-transformed index shows a tighter distribution with fewer extreme values, indicating reduced impact of outliers.
(C) Normal Q–Q plot for the TG/UA ratio, showing significant upward deviation from the reference line in the upper quantiles, confirming right-skewness and non-normality.
(D) Normal Q–Q plot for the TG–UA index, where data points closely adhere to the reference line with minimal tail deviations, demonstrating that the transformation effectively normalizes the distribution and mitigates the impact of outliers.

**References**

[1] Azur MJ, Stuart EA, Frangakis C, et al. Multiple imputation by chained equations: what is it and how does it work? Int J Methods Psychiatr Res 2011; 20: 40–49.

[2] Nguyen CD, Carlin JB, Lee KJ. Diagnosing problems with imputation models using the Kolmogorov-Smirnov test: a simulation study. BMC Med Res Methodol 2013; 13: 144.

[3] Shrestha N. Detecting Multicollinearity in Regression Analysis. AJAMS 2020; 8: 39–42.

[4] Leeuwenberg AM, Van Smeden M, Langendijk JA, et al. Performance of binary prediction models in high-correlation low-dimensional settings: a comparison of methods. Diagn Progn Res 2022; 6: 1.

[5] Chan JY-L, Leow SMH, Bea KT, et al. Mitigating the Multicollinearity Problem and Its Machine Learning Approach: A Review. Mathematics 2022; 10: 1283.

[6] Davino C, Romano R, Vistocco D. Handling multicollinearity in quantile regression through the use of principal component regression. METRON 2022; 80: 153–174.

[7] Arévalo-Cordovilla FE, Peña M. Evaluating ensemble models for fair and interpretable prediction in higher education using multimodal data. Sci Rep 2025; 15: 29420.

[8] Huang Y, Song X, Chen Y, et al. Intratumoral Microbiome–related MRI Model for Predicting Breast Cancer Shrinkage Pattern Following Neoadjuvant Therapy. Radiology 2025; 316: e243545.

[9] Tibshirani R. Regression Shrinkage and Selection Via the Lasso. J R Statist Soc 1996; 58: 267–288.

[10] Akiba T, Sano S, Yanase T, et al. Optuna: A Next-generation Hyperparameter Optimization Framework. In: Proceedings of the 25th ACM SIGKDD International Conference on Knowledge Discovery & Data Mining. Anchorage AK USA: ACM, pp. 2623–2631.
