# Supplementary material for: Preoperative Metabolic Predictors of Granulation Subtypes in Somatotroph Tumors: A Multicenter Retrospective Cohort Study
Source: CNS Neurosci Ther. 2026 Feb 3;32(2):e70774. doi: 10.1002/cns.70774 (PMC12865497; doi:10.1002/cns.70774)
Supplement: Supplementary file 2 — Table S1: Summary statistics of the raw TG/UA ratio and its log‐transformed TG–UA index. Table S2: Detailed description of variables and coding schemes. Table S3: Comparison of DGST and SGST patients in Center A and the combined cohort (Centers A–C). Table S4: Principal component loadings and explained variance for lipid and tumor morphology variables. Table S5: Subgroup analysis of UA, TG, and the TG–UA Index. [file CNS-32-e70774-s002.docx]

**Pre-operative Metabolic Predictors of Granulation Subtypes in Somatotroph Tumors: A Multicenter Retrospective Cohort Study**

***Supplementary Tables***

**Table S1. Summary statistics of raw TG/UA ratio and its log-transformed TG–UA index**

| **Metric** | **TG/UA Ratio** | **TG–UA Index** |
| --- | --- | --- |
| Mean ± SD | 5.43 ± 4.15 | 1.49 ± 0.61 |
| Median [IQR] | 4.31 [2.88–6.71] | 1.46 [1.06–1.90] |
| Skewness | 2.85 | 0.40 |
| Shapiro–Wilk p | 5.34e−19 | 0.019 |

*Note:* Compared with the raw TG/UA ratio, the TG–UA index shows markedly reduced skewness and dispersion, as well as an attenuated deviation from normality, as reflected by the higher Shapiro–Wilk p value.

Abbreviations: TG, triglyceride; UA, uric acid; SD, standard deviation; IQR, interquartile range.

**Table S2. Detailed description of variables and coding schemes**

| **Variable** | **Type** | **Coding scheme / Unit** | |
| --- | --- | --- | --- |
| Age | Continuous | years |  |
| Sex | Categorical | female=0, male=1 |  |
| Height | Continuous | cm |  |
| Weight | Continuous | kg |  |
| BMI | Continuous | kg/m² |  |
| Hypertension | Categorical | without=0, with =1 |  |
| Diabetes | Categorical | without=0, with =1 |  |
| Duration of disease | Continuous | months |  |
| UA | Continuous | μmol/L |  |
| TG | Continuous | mmol/L |  |
| TC | Continuous | mmol/L |  |
| LDL-C | Continuous | mmol/L |  |
| GH | Continuous | μg/L |  |
| IGF-1 | Continuous | ng/mL |  |
| Knosp grade | Categorical | grades 0–4 |  |
| Maximum transverse diameter | Continuous | mm |  |
| Maximum anteroposterior diameter | Continuous | mm |  |
| Maximum superoinferior diameter | Continuous | mm |  |
| Tumor volume | Continuous | cm³ |  |
| Center | Categorical | A=1, B=2, C=3 |  |
| Sparse-dense granule pattern | Categorical | DGST=0, SGST=1 |  |

*Note:* BMI was included as a covariate in all analyses to represent overall adiposity and metabolic status. Height and weight were not entered as separate predictors.

Abbreviations: BMI, body mass index; UA, uric acid; TG, triglyceride; TC, total cholesterol; LDL-C, low-density lipoprotein cholesterol; GH, growth hormone; IGF-1, insulin-like growth factor 1.

**Table S3****. Comparison of DGST and SGST patients in Center A and the combined cohort (Centers A–C)**

| **Variables** | **A** | | **p** | | **A–C** | | **p** |
| --- | --- | --- | --- | --- | --- | --- | --- |
|  | **DGST(n=91)** | **SGST(n=86)** |  | | **DGST(n=121)** | **SGST(n=109)** |  |
| Duration of disease | 24.0 (12.0, 60.0) | 24.0 (10.3, 60.0) | | 0.934 | 24.0 (6.0, 60.0) | 24.0 (5.0, 60.0) | 0.796 |
| Hypertension |  |  | |  |  |  |  |
| With | 20(22.0%) | 16(18.6%) | | 0.711 | 23(19.0%) | 20(18.3%) | 1.000 |
| Diabetes |  |  | |  |  |  |  |
| With | 20 (22.0%) | 18 (20.9%) | | 1.000 | 23 (19.0%) | 22 (20.2%) | 0.954 |
| TC | 4.5 (3.8, 5.2) | 4.7 (4.3, 5.2) | | 0.062 | 4.5 (4.0, 5.2) | 4.6 (4.2, 5.0) | 0.274 |
| Knosp grade |  |  | |  |  |  |  |
| 0 | 19 | 7 | |  | 21 | 8 |  |
| 1 | 29 | 27 | |  | 40 | 32 |  |
| 2 | 20 | 20 | |  | 29 | 27 |  |
| 3 | 12 | 16 | |  | 17 | 21 |  |
| 4 | 11 | 16 | | 0.137 | 14 | 21 | 0.091 |

Abbreviations: A, Center A; A–C, Centers A–C; TC, total cholesterol.

**Table S4. Principal component loadings and explained variance for lipid and tumor morphology variables**

| 1. **Lipid Variables** | **Lipid_PC1** | **Lipid_PC2** |
| --- | --- | --- |
| TC | 0.707 | 0.707 |
| LDL-C | 0.707 | -0.707 |
| **Explained variance (%)** | **96.64%** | **3.36%** |

Abbreviations: TC, total cholesterol; LDL-C, low-density lipoprotein cholesterol.

| 1. **Tumor Morphology Variables** | **Tumor_PC1** | **Tumor_PC2** | **Tumor_PC3** | **Tumor_PC4** |
| --- | --- | --- | --- | --- |
| Maximum transverse diameter | 0.500 | 0.018 | -0.842 | 0.200 |
| Maximum anteroposterior diameter | 0.493 | -0.804 | 0.215 | -0.253 |
| Maximum superoinferior diameter | 0.501 | 0.553 | 0.156 | -0.647 |
| Tumor volume | 0.505 | 0.218 | 0.469 | 0.691 |
| **Explained variance (%)** | **85.95%** | **5.65%** | **4.70%** | **3.71%** |

**Table S5. Subgroup analysis of UA, TG, and the TG–UA Index**

| **Variable** | **Stratification variable** | **OR** | **95% CI** | **p** | **p for interaction** |
| --- | --- | --- | --- | --- | --- |
| UA |  |  |  |  |  |
|  | Sex |  |  |  | 0.494 |
|  | Male | 0.856 | 0.532-1.378 | 0.523 |  |
|  | Female | 0.791 | 0.508-1.231 | 0.298 |  |
|  | Age |  |  |  | 0.985 |
|  | <=40 | 1.006 | 0.640-1.582 | 0.979 |  |
|  | >40 | 0.621 | 0.378-1.020 | 0.060 |  |
|  | Diabetes |  |  |  | 0.763 |
|  | With | 0.423 | 0.112-1.595 | 0.204 |  |
|  | Without | 0.826 | 0.582-1.171 | 0.282 |  |
|  | Hypertension |  |  |  | 0.080 |
|  | With | 0.113 | 0.020-0.639 | 0.014 |  |
|  | Without | 0.939 | 0.664-1.329 | 0.723 |  |
| TG |  |  |  |  |  |
|  | Sex |  |  |  | 0.883 |
|  | Male | 1.253 | 0.801-1.962 | 0.323 |  |
|  | Female | 1.545 | 0.889-2.687 | 0.123 |  |
|  | Age |  |  |  | 0.550 |
|  | <=40 | 1.379 | 0.821-2.315 | 0.224 |  |
|  | >40 | 1.614 | 0.981-2.656 | 0.060 |  |
|  | Diabetes |  |  |  | 0.460 |
|  | With | 0.724 | 0.170-3.079 | 0.662 |  |
|  | Without | 1.529 | 1.032-2.266 | 0.034 |  |
|  | Hypertension |  |  |  | 0.414 |
|  | With | 2.008 | 0.843-4.786 | 0.116 |  |
|  | Without | 1.275 | 0.872-1.865 | 0.211 |  |
| TG–UA index |  |  |  |  |  |
|  | Sex |  |  |  | 0.691 |
|  | Male | 1.322 | 0.806-2.168 | 0.268 |  |
|  | Female | 1.741 | 1.066-2.843 | 0.027 |  |
|  | Age |  |  |  | 0.971 |
|  | <=40 | 1.376 | 0.850-2.226 | 0.194 |  |
|  | >40 | 1.941 | 1.150-3.277 | 0.013 |  |
|  | Diabetes |  |  |  | 0.412 |
|  | With | 1.51 | 0.383-5.963 | 0.556 |  |
|  | Without | 1.68 | 1.161-2.432 | 0.006 |  |
|  | Hypertension |  |  |  | 0.069 |
|  | With | 5.19 | 1.372-19.629 | 0.015 |  |
|  | Without | 1.322 | 0.915-1.911 | 0.137 |  |

Abbreviations: OR, odds ratio; 95% CI, 95% confidence interval; UA, uric acid; TG, triglyceride.
